# Deoxyarbutin displays antitumour activity against melanoma *in vitro* and *in vivo* through a p38-mediated mitochondria associated apoptotic pathway

**DOI:** 10.1038/s41598-017-05416-8

**Published:** 2017-08-03

**Authors:** Limei Ma, Yao Xu, Zeliang Wei, Guang Xin, Zhihua Xing, Hai Niu, Wen Huang

**Affiliations:** 1 Laboratory of Ethnopharmacology/Regenerative Medicine Research Center, West China Hospital, Sichuan University, Chengdu, Sichuan 610041 China; 2 College of Mathematics, Sichuan University, Chengdu, Sichuan 610041 China

## Abstract

Deoxyarbutin (DeoxyArbutin, dA), a natural compound widely used in skin lighting, displayed selectively cytotoxicity *in vitro*. In the study, we found that dA significantly inhibited viability/proliferation of B16F10 melanoma cells, induced tumour cell arrest and apoptosis. Furthermore, dA triggered its pro-apoptosis through damaging the mitochondrial function (membrane potential loss, ATP depletion and ROS overload generation etc.) and activating caspase-9, PARP, caspase-3 and the phosphorylation of p38. Treatment with p38 agonist confirmed the involvement of p38 pathway triggered by dA in B16F10 cells. The *in vivo* finding also revealed that administration of dA significantly decreased the tumour volume and tumour metastasis in B16F10 xenograft model by inhibiting tumour proliferation and inducing tumour apoptosis. Importantly, the results indicated that dA was specific against tumour cell lines and had no observed systemic toxicity *in vivo*. Taken together, our study demonstrated that dA could combate tumour *in vitro* and *in vivo* by inhibiting the proliferation and metastasis of tumour via a p38-mediated mitochondria associated apoptotic pathway.

## Introduction

Cutaneous melanoma is one of the most lethal and fastest growing forms of human cancers to affect a younger population^1, 2^. Despite advances in surgery and multi-agent chemotherapy, once the tumour advanced, nearly 80% of patients still die from metastatic melanoma^3^. The survival rates for metastatic melanoma remain relatively low to 6–9 months compared to lung cancer (the leading cause of cancer-related deaths worldwide with 4–10 months survival time)^4, 5^. Surgical resections including BRAF inhibitors (the first-line therapy for melanoma treatment), interleukin-2 biological therapy show various drawbacks, such as toxicities, unsatisfactory efficacy and rapid development of resistance^6–8^. Therefore, it is urgent to develop novel therapeutic options with low side effects on melanoma treatment.

Intervention of natural products in cancer growth and progression has become very popular. And, statistically about 36% of the small molecule compounds approved by Food and Drug Administration (FDA) are natural products or their derivatives^9^. In addition, a large body of epidemiological studies have verified that the natural factors, including resveratrol, lycopene, dioscin and polyunsaturated omega-3 fatty acids (PUFA), play an indispensable role in preventing cancers cell lines with lower toxicities^10–14^.

**Figure 1:**
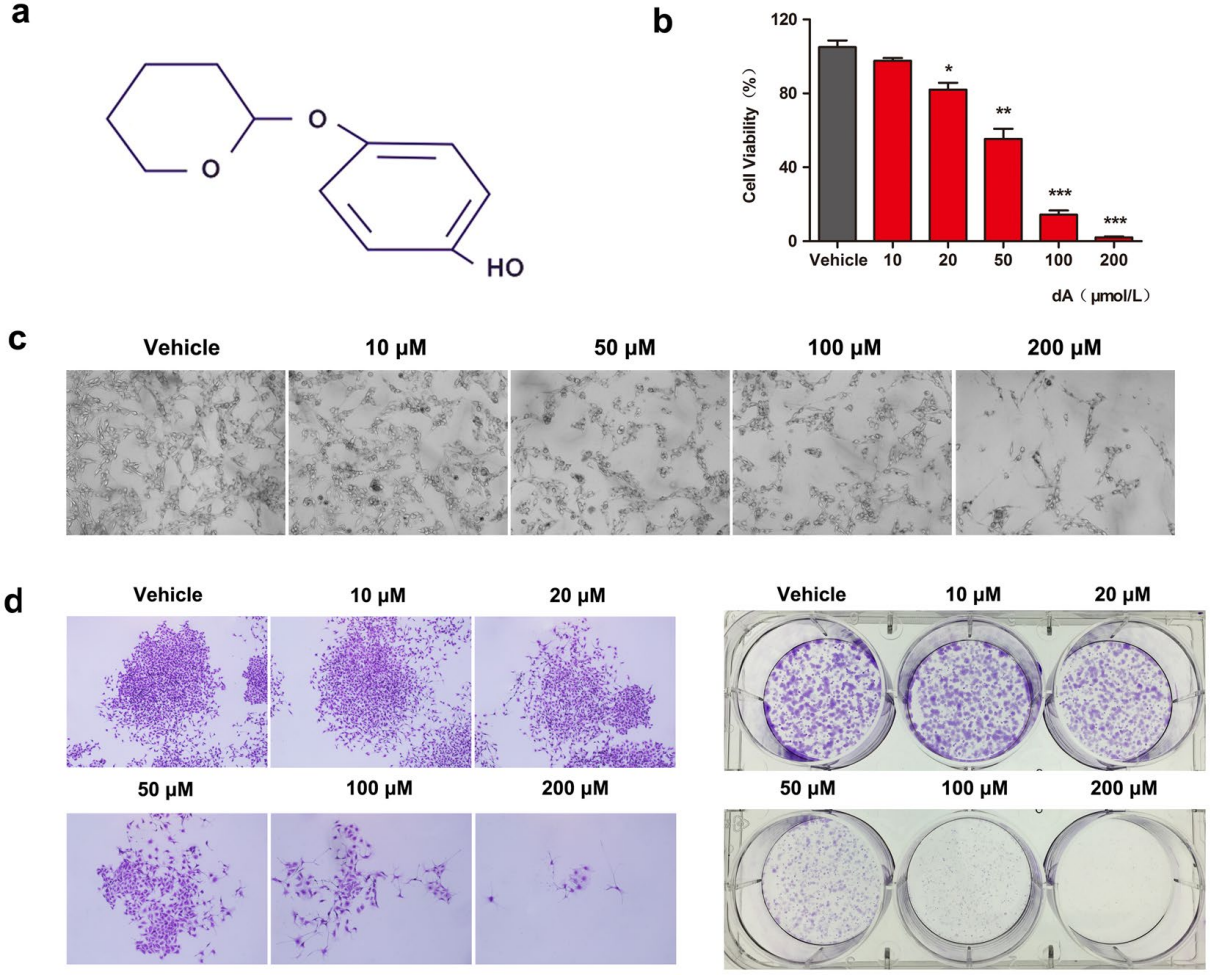
dA inhibited proliferation of B16F10 cells in a concentration dependent manner. (**a**) Structure of dA (4-[(tetrahydro-2H-pyran-2-yl) oxy] phenol). (**b**) Cell viability was determined by CCK-8 assay after 24 h treatment with different concentrations of dA (10, 20, 50, 100 and 200 μM). Vehicle indicated cells without treating dA. (**c**) Morphologic measurements in B16F10 cells after treating with various concentration of dA for 24 h. (**d**) Colony formation was carried out via crystal violet staining. The data represent mean ± s.d. of the three independent experiments. *p < 0.05, **p < 0.01 and ***p < 0.001 compared with the Vehicle group.

Deoxyarbutin (4-[(tetrahydro-2H-pyran-2-yl) oxy] phenol, dA) (Fig. 1a), a commercial product in skin lightening, appears to have similar activities as hydroquinone (1, 4-benzenediol, HQ)^15–17^. Previous studies have demonstrated HQ could inhibit tyrosine activity as well as induce DNA damage via generation of reactive oxygen species (ROS)^18^. Wang *et al*. showed HQ could induce apoptosis and result in cytotoxicity in mouse primary hepatocytes, which could be reserved by resveratrol^19^. Hydroxyl hydroquinone (HHQ), also obtained from HQ, was observed to decrease cell viability and colony formation by induction of oxidative stress that leads to apoptosis^20^. dA was safer and less cytotoxic compared with HQ and HHQ^15, 20^. Miao *et al*. revealed that dA possessed a potent skin lightening ability by regulating ROS generation with less melanosome cytotoxicity *in vitro* and *in vivo* models^21^. However, studies on the pro-apoptotic effect of this bioactive compound on cancer cells are limited.

Induction of apoptosis in cancer cells is one of key approaches for cancer therapy^22, 23^. Evidence demonstrates mitochondria are major intracellular sites responsible for energy metabolism of growth and proliferation. Apoptosis in cancer cells could be closely linked with mitochondrial dysfunction^24–26^. MAP kinase family, especially involvement of p38, has been implicated in mitochondrial dysfunction and apoptotic pathway^26–28^. Several chemotherapeutic agents, such as nocodazole, vinblastine as well as taxol could activate p38 MAPK pathway and induce cell cycle arrest by affecting mitochondrial damage and ROS gerneation^29^. Also p38 and its mediated signaling are critical targets to answer ROS stress^30–32^. Together, these findings suggest the potential role of p38 MAPK in anticancer therapy concerning mitochondria associated apoptotic pathway.

In the present work, we reported that dA exhibited antitumour effect against melanoma *in vitro* and *in vivo*. dA induced the apoptotic death of tumour cells by initiating a p38 mediated mitochondria associated pathway. Our results firstly provide the evidence that dA could be an alternative agent with satisfying therapeutic efficacy on the treatment of melanoma.

## Results

### dA selectively inhibited B16F10 cell proliferation with low cytotoxicity in normal cell lines

The effect of dA on the proliferation of various cell lines was determined by using a Cell Counting Kit-8 (CCK-8) or MTT (3-(4,5-dimethyl-2-thia-zolyl)-2, 5-diphenyl-2H-tetrazolium bromide) assay. Cell viability was separately evaluated in six normal cell lines (NIH/3T3, HS68, HK-2, L02, HLECs and HUVECs cells) and two cancer cells (B16F10, LL/2), which had been treated with varying concentrations from 10 μM to 200 μM of dA for 24 h. Results showed that dA significantly inhibited the proliferation of tumour cells including B16F10 cells and LL/2 cells, especially B16F10 cells (EC50 = 39.56 μM). While intervened with other normal cell lines, dA displayed lower cytotoxicity as shown in Fig. 1b, Supplementary Figs 1 and 2. Moreover, results of colony formation assay revealed that the size of the colony formation treated with dA was significantly smaller; and the number of melanoma cells significantly decreased in a concentration dependent manner (Fig. 1c,d), indicating that dA could selectively suppress the proliferation of B16F10 cells with low side effects on normal cell lines.

### dA induced cell cycle arrest and motivated apoptosis in B16F10 cells

**Figure 2:**
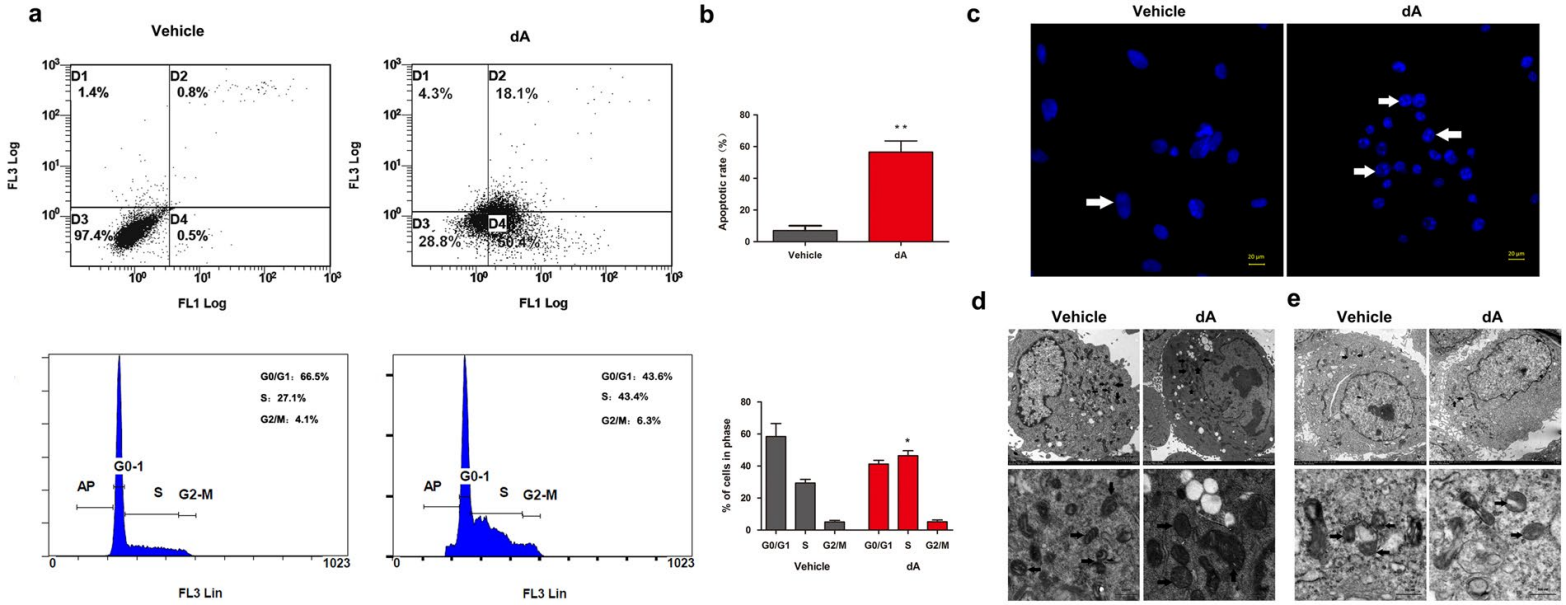
dA induced cell cycle arrest and motivated apoptosis in B16F10 cells. (**a**) Number of apoptosis cells and DNA cell cycle rate in B16F10 cells was analyzed by using flow cytometry after treating with 50 μM dA for 24 h. Vehicle indicated cells without treating dA. (**b**) The apoptotic rates and cycle arrest rate of B16F10 cells after dA treatment. (**c**) Morphologic changes in B16F10 cells were examined by using DAPI fluorescence staining. (**d**,**e**) TEM images showing the microcosmic changes after treatment 50 μM of dA in B16F10 cells (**d**) and NIH3T3 cells (**e**). Lower panel indicated the local mitochondrial microcosmic changes in both groups. Data are presented as mean ± s.d. of the three independent experiments. *p < 0.05, **p < 0.01 compared with the Vehicle group.

DNA cell cycle and apoptotic rate analysis were used to examine the effect of dA on proliferation of B16F10 cells. Based on the EC50 value and more than 50% of viable cell number after dA treatment, 50 μM was employed as the optimal concentration for treatment in the research. As shown in Fig. 2a,b, the S phase significantly increased from 27.10% to 43.40%, while the number of cells in the G0/G1 phase gradually decreased from 66.50% to 43.60% after B16F10 cells were incubated with dA for 24 h. Also, the percentage of late apoptotic cells increased to 15.35%, while the percentage of early apoptotic cells increased to 50.36% following treatment with 50 μM dA, as compared with the untreated group. The data suggest that dA triggered cell cycle arrest and induced apoptosis in B16F10 cells (*P* < 0.05). Meanwhile, we also evaluated the cell cycle arrest and apoptotic situation in NIH/3T3 and HS68 cells treated with dA. As shown in Supplementary Fig. 3a,b, dA treatment did not induce detectable apoptosis and cell arrest compared with the vehicle group, suggesting that dA could selectively play a pro-apoptotic role and inhibit proliferation in B16F10 cells.

Morphological changes were also observed in B16F10 cells following treatment with dA for 24 h (Fig. 2c). Cells in dA-treated group exhibited apoptotic features such as shrinkage, chromatin congregation and nuclear fragmentation compared with the dA-untreated cells. Meanwhile, a transmission electronic microscope (TEM) was used to identify morphological changes in the ultrastructure of the B16F10 cells. The results in Fig. 2d showed that cells treated with dA appeared chromatin congregation and cytoplasm shrinkage such ultrastructural changes, while no clear damage was observed in the vehicle group. We also employed a normal cell line NIH/3T3 to confirm the specificity of dA in melanoma cancer cells. As shown in Fig. 2e, dA treatment did not produce morphological changes related to apoptosis in NIH/3T3 cells, suggesting that dA could selectively induce apoptosis in B16F10 cells. Interestingly, it also may be noted mitochondrial swelling in dA-treated group in B16F10 cells rather than NIH/3T3 cells (Fig. 2d,e), implying that the apoptosis of B16F10 cells induced by dA was related to mitochondria.

### dA induced cell death related to the mitochondrial dysfunction

**Figure 3:**
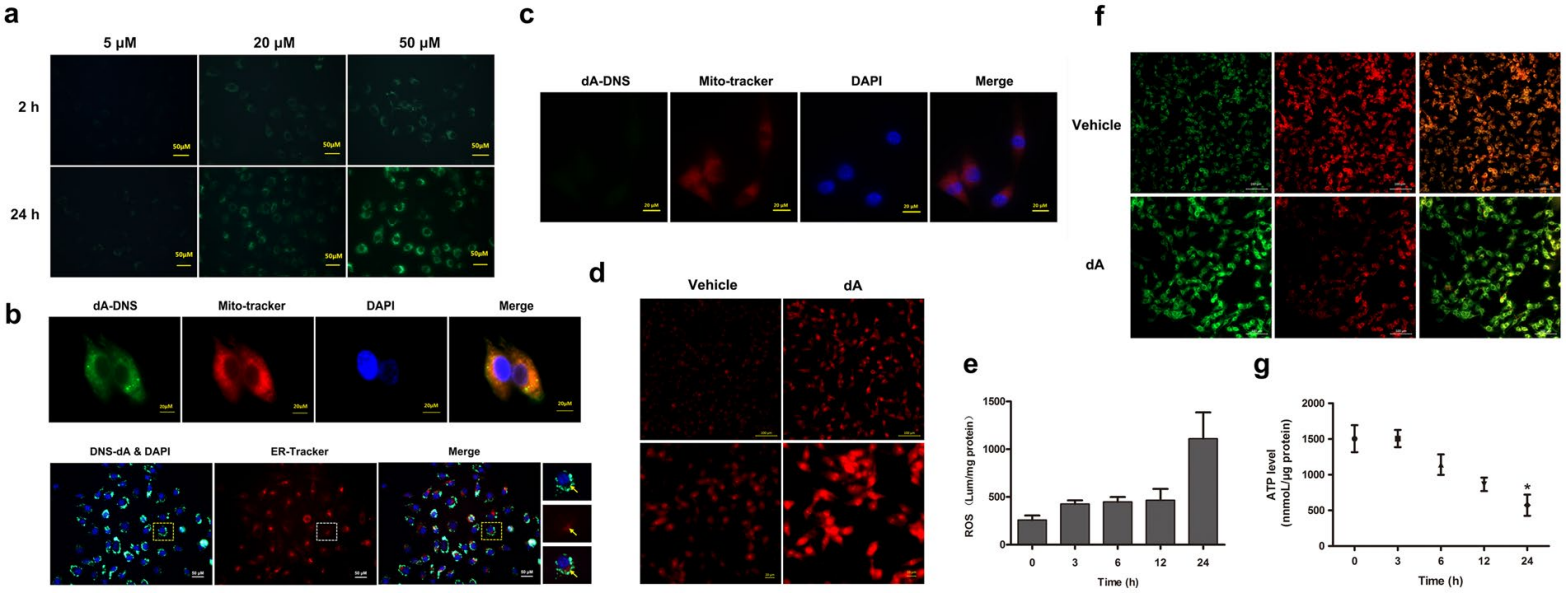
dA induced cell death related to mitochondrial dysfunction in B16F10 cell line. (**a**) Confocal microscopy for DNS-labled-dA treatment for 2 h and 24 h vary concertation from 20 μM to 50 μM. (**b**,**c**) Co-localization of DNS-dA (green) and Mito-Tracker (red) in B16F10 cells (**b**) and NIH/3T3 cells (**c**). Cells were incubated in 50 μM DNS-dA for 24 h and stained with Mito-Tracker for another 15 min. Confocal microscopy image revealed that dA selectively penetrated into B16F10 cells other than NIH/3T3 cells. B16F10 cells were also stained with ER-tracker (red) followed 50 μM DNS-dA’s treatment. DAPI (blue) was used for nuclei detection. Confocal microscopy image showed that dA was not co-localization with ER-Tracker. The yellow arrows refer to the cells within indicated boxes. Images were representative of 3 independent experiments. (**d**,**e**) The confocal microscopy image of ROS levels in B16F10 cells after 24 h treatment staining by mito-sox. The relative fluorescent intensity of intracellular ROS levels after dA treatment for different time. (**f**) The representative images of mitochondrial membrane potential determined by confocal microscopy. The cells with green-positive and red-negative fluorescence were counted as depolarized cells. (**g**) The relative ATP levels indicated by protein content. Data are presented as the mean ± s.d. of three independent experiments. *p < 0.05 VS the Vehicle group.

Mitochondria are one of the major site responsible for cell apoptosis as well as production of ROS^33, 34^. To further evaluate the mitochondrial function in dA’ pro-apoptotic activity, we applied dansyl chloride (DNS) to lable dA, and then incubated with B16F10 cells in time course. The results showed that DNS-labeled-dA rapidly penetrated into cytomembrane and enriched in cytoplasm in a time- and concentration- dependent manners (Fig. 3a). Using TEM, we found that dA led to a mitochondrial damage in tumour cells specificly. Results of confocal microscope also showed that dA was co-localized with mitochondria but no endoplasmic reticulum (ER) in B16F10 cells after treating for 24 h (Fig. 3b), suggesting that dA mainly internalize into mitochondria and then trigger mitochondrial damage. In addition, the results shown in Fig. 3c indicated that exposure of NIH/3T3 cells to DNS-dA showed no specific immunofluorescence co-localization of Mito-tracker and DNS-dA. Together, our data confirmed that dA could specifically orientate cancer cells and induce mitochondrial dysfunction in B16F10 cells.

ROS overload is the leading cause to induce the deterioration of functional and structural integrity of the mitochondria. ROS, ATP and membrane potential were considered as the preferred detection index for evaluating mitochondria function^35^. As shown in Fig. 3g, there was a time-dependent decrease in intracellular ATP content in dA-treated cells, and low to 25% of its control value at 24 h. An increase in ROS formation was also observed after cells were incubated with dA for 3 h, which significantly arose at 24 h (4 folds compared with the cells without treating dA at 0 h) (Fig. 3e). Confocal microscopic images also confirmed that dA induced a remarkable increase of ROS level at 24 h (Fig. 3d). In addition, it may be noted from confocal microscopic images that dA depolarize the inner mitochondrial membrane potential after treating cells with 24 h (Fig. 3f). Altogether, these data showed that dA could internalize into mitochondria, and then caused mitochondrial membrane potential loss, ROS generation and ATP depletion.

### dA regulated the expression of mitochondria associated apoptotic proteins

**Figure 4:**
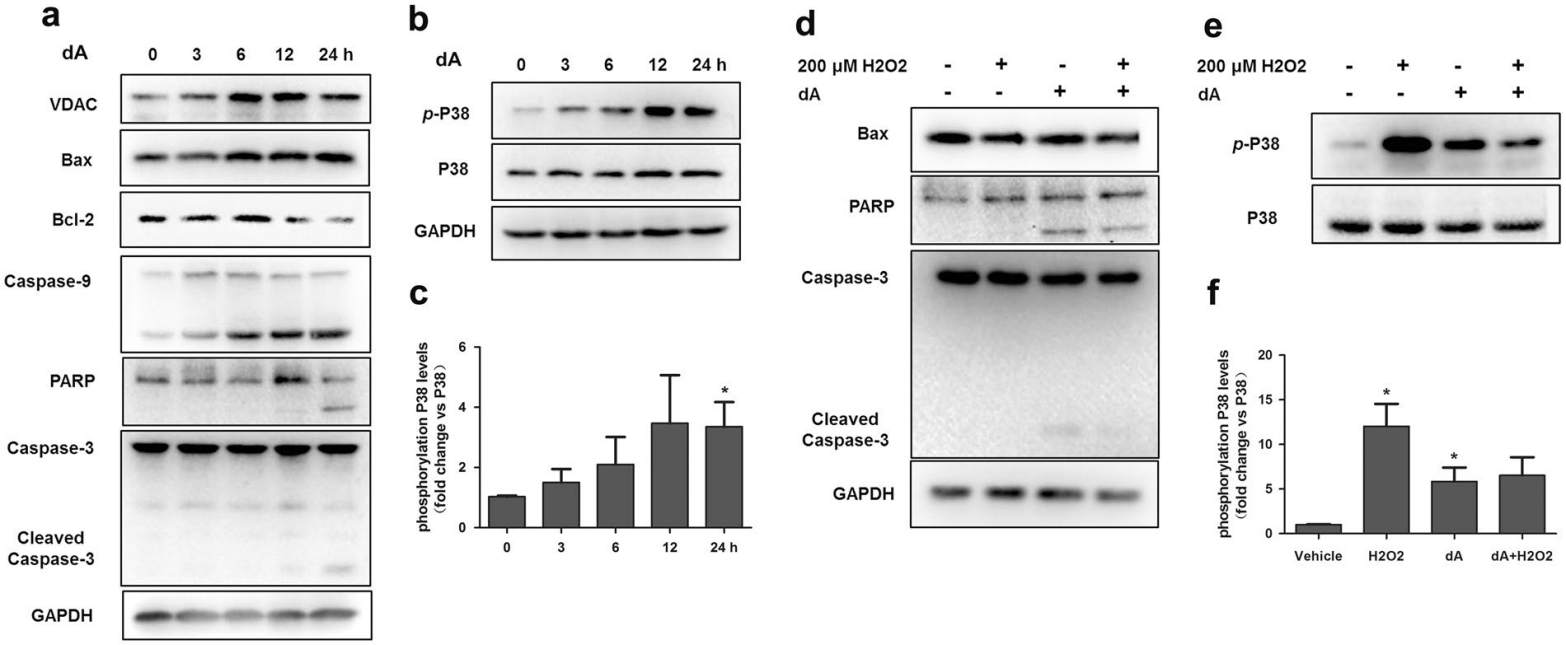
dA regulated the expression of mitochondria associated apoptotic proteins. (**a**) Western blot analysis of mitochondria associated apoptotic proteins expression after dA treatment for different time (3 h, 6 h, 12 h, 24 h). Treating for 0 h was used as the internal standard; (**b**,**c**) Expression of phosphorylated p38 (p-p38), as measured by Western blot. Lower panel: densitometry analysis of p-p38 expression (n = 3) *p < 0.05 vs 0 h. Data are expressed as the fold change over p38 (set as 1). *p < 0.05 vs the respective control. (**d**,**e**,**f**) B16F10 cells were incubated with 50 μM dA for 24 h and then treated with 200 μM H_2_O_2_ for 2 h. (**d**) Western blot analysis of Bax, PARP, and Caspase-3 expression. GAPDH were used as internal standards (n = 3). (**e**) Expression of p-p38 after treating with dA and H_2_O_2_. (**f**) The fold changes of p-p38 expression over the respective control. The results obtained from 3 independent experiments. *p < 0.05, **p < 0.01 vs control group (0 h). Full-length blots indicated in Fig. 4 were presented in Supplementary Fig. 4.

Since caspase-9/3 and Bcl family are important regulators of mitochondria associated apoptosis^36, 37^, we investigated their involvement during dA treatment in B16F10 cells. Figure 4a and Supplementary Fig. 4a showed that dA decreased the expression of Bcl-2 and increased the expression of Bax in a time-dependent manner. Caspase-9 and caspase-3 were also cleaved and accumulated following dA treatment at 24 h. We further verified whether dA could activate PARP, a cellular protein cleaved by caspase-3 and associated with apoptosis^38^. It was observed that caspase-9 activation in B16F10 cells treated with dA was happened at 6 h, while caspase-3 activation at 12 h and arrived at plateau at 24 h. The cleaved substrate of caspase-3, PARP, could be also observed from 12 h to 24 h. The data suggest that dA induced apoptosis by activating caspase-9, and regulating PARP cleaved, finally leading to caspase-3 activation. We also found the expression of VDAC, another mitochondria associated apoptotic protein regulating by translocation of Bax from cytosol into mitochondria^39, 40^, was upregulated upon treatment with dA at 24 h compared with 0 h, suggesting that dA could active VDAC just the same as Bax and involved in the mitochondrial associated apoptosis. These data further confirmed that dA inhibited Bcl-2, activated Bax and VDAC, which in turn sequentially activated caspase-9, PARP, caspase-3 and finally led to mitochondria associated apoptosis.

Moreover, the MAP kinase family, especially p38, has been implicated in mitochondria associated apoptosis^26–29^. As shown in Fig. 4b,c and Supplementary Fig. 4a, phosphorylated p38 (p-p38) was detected by western blot at 12 h after stimulation by 50 μM dA, and it maintained a plateau of activation after 24 h. H_2_O_2_ is known to be an agonist of p-p38^41^. The results from Fig. 4e,f revealed that H_2_O_2_ could efficiently enhance expression of p-p38 as reported in literatures^41^. dA did induce the augmented p-p38 at protein level as 200 μM H_2_O_2_ did. While cells were co-treated with dA (a stonger antioxidant) and H_2_O_2_ (one of the strongest oxidants), both dA and H_2_O_2_ reacted chemically and consumed either H_2_O_2_ or dA, leading to lower p-p38 levels in dA + H_2_O_2_ group, compared with that in 200 uM H_2_O_2_ alone treated group, which further implying the involvement of p38 pathway triggered by dA in B16F10 cells. In addition, results in Fig. 4d and Supplementary Fig. 4b also showed dA induced apoptosis regardless of the presence or absence of H_2_O_2_, and H_2_O_2_ didn’t enhance pro-apoptotic activity of dA in the experimental condition. Together, our study indicated that dA displayed a remarkable pro-apoptotic activity mainly due to the involvement of p38 MAPK pathway.

### dA suppressed melanoma tumour growth related to a p38 mediated mitochondria associated apoptosis *in vivo*

**Figure 5:**
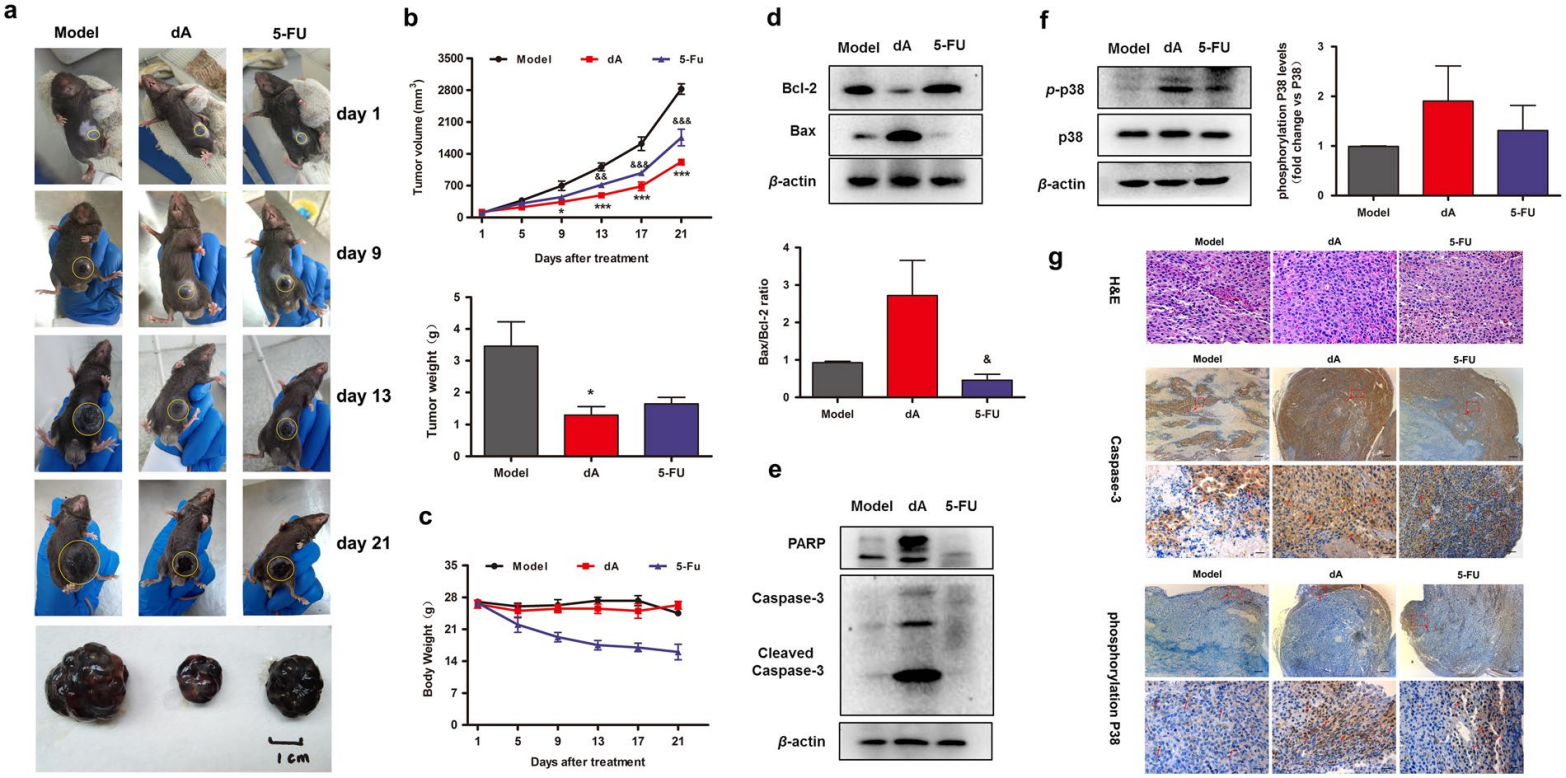
dA suppressed melanoma tumour growth related to mitochondria associated apoptosis *in vivo*. Mice were subcutaneously injected with 1.0 × 10^6^ B16F10 cells and the antitumour effect of 50 mg/kg dA or 5-FU (30 mg/kg) was evaluated. Saline was used as the Vehicle control, once a day for 3 weeks. (**a**) Typical photographs taken at different time-points and examined the growth of subcutaneous melanomas, as indicated by yellow circles. (**b**) Tumour growth monitored at different time-points and volume calculation. Lower panel: Data represent the excised tumours weight from 5–10 animals per experimental group at 21 day. (**c**) Percentage body weight per experimental group at indicated time point. (**d**) Western blot analysis of pro-apoptosis protein Bax and anti-apoptosis protein Bcl-2 in tumour sections obtained from 5–10 animals per experimental group. (**e**) Western blot analysis of mitochondria associated apoptotic proteins expression in tumour sections excised from mice at day 21 of treatment. (**f**) Expression of phosphorylated p38 (p-p38) per experimental group in tumour sections, as measured by Western blot. Lower panel: densitometry analysis of p-p38 expression and data are expressed as the fold change over p38 (set as 1). The full-length blots were presented in Supplementary Fig. 5a. (**g**) H&E stained tumour sections of mice from treated and vehicle sets to evaluate the effect of dA on cell morphology. The expression of active caspase-3 and phosphorylated p38 per experimental group, Scale bar: 100 μm. Lower panel: Protein expression in red box area above, scale bar: 20 μm. Images represent the results obtained from 3 animals per experimental group. Images and data represent the results obtained from 3 animals per experimental group. *p represented dA group, ^&^ *p* represented 5-FU group vs the respective Vehicle group. *p < 0.05, **p < 0.01, ***p < 0.001 as well as ^&^ *p*.

Here, we further validated the *in vivo* activity against tumour by a subcutaneously grafted murine melanoma model. As shown in Fig. 5a,b, the average tumour size in the dA-and 5-Fluorouracil (5-FU) treated groups were 494.91 ± 114.10 and 720.90 ± 31.32 mm^3^ respectively. Whereas the average tumour size in the model group was 1122.91 ± 284.13 mm^3^. The results indicated that treatment of dA decreased tumour volumes more effective than 5-FU did. Tumour weight of the dA- and 5-FU-treated group as shown in Fig. 5b were also significantly reduced respectively compared with model group. The data proposed that dA exhibited an efficient inhibition of tumour growth than 5-FU, one of the standard clinical strategy for patients with malignant tumour.

Body weight fluctuation of mice was also an antitumour indicator measured over the course of the treatment period. No significant difference was observed in dA-treated group; however, the body weights of the mice in the 5-FU-treated group were decreased compared with the model group (Fig. 5c). The results suggest that dA with lower side effects was more effective than 5-FU in antitumour treatment.

In agreement with the *in vitro* results, western blot experiments revealed a suppression of Bcl-2 expression, accompanied with an increasing of Bax expression in melanomas treated with dA, leading to a raise in the Bax/Bcl-2 ratio as shown in Fig. 5d and Supplementary Fig. 5a. Also, the active expressions of PARP, caspase-3 and phospho-p38 were enhanced in dA-treated group (Fig. 5e,f and Supplementary Fig. 5a). While, 5-FU in dose of less than 30 mg/kg *in vivo* wasn’t observed to stimulate apoptotic proteins including Bax, PARP, caspase-3, suggesting that dA was more effective than 5-FU in producing apoptosis of tumour in the experimental condition. In the present study, we have found that 5-FU in dose of more than 40 mg/kg could directly result in higher mortality of mice, though 5-FU was observed to induce the expressions of Bax, PARP cleavage and cleaved caspase-3. These results suggest that the expression of apoptotic proteins are highly related to the dose of 5-FU. In addition, immunostaining experiments of the cleaved caspase-3 and phospho-p38 revealed a higher amount of clustered apoptotic cells in tumour sections treated with dA compared with model group (Fig. 5g and Supplementary Fig. 5a). Together, these data indicated that the antitumour effect of dA was more valid than 5-FU and closely related to p38 mediated mitochondria associated apoptosis.

### dA inhibited melanoma B16F10 cell lung metastasis related to p38 mitochondria associated apoptosis *in vivo*

**Figure 6:**
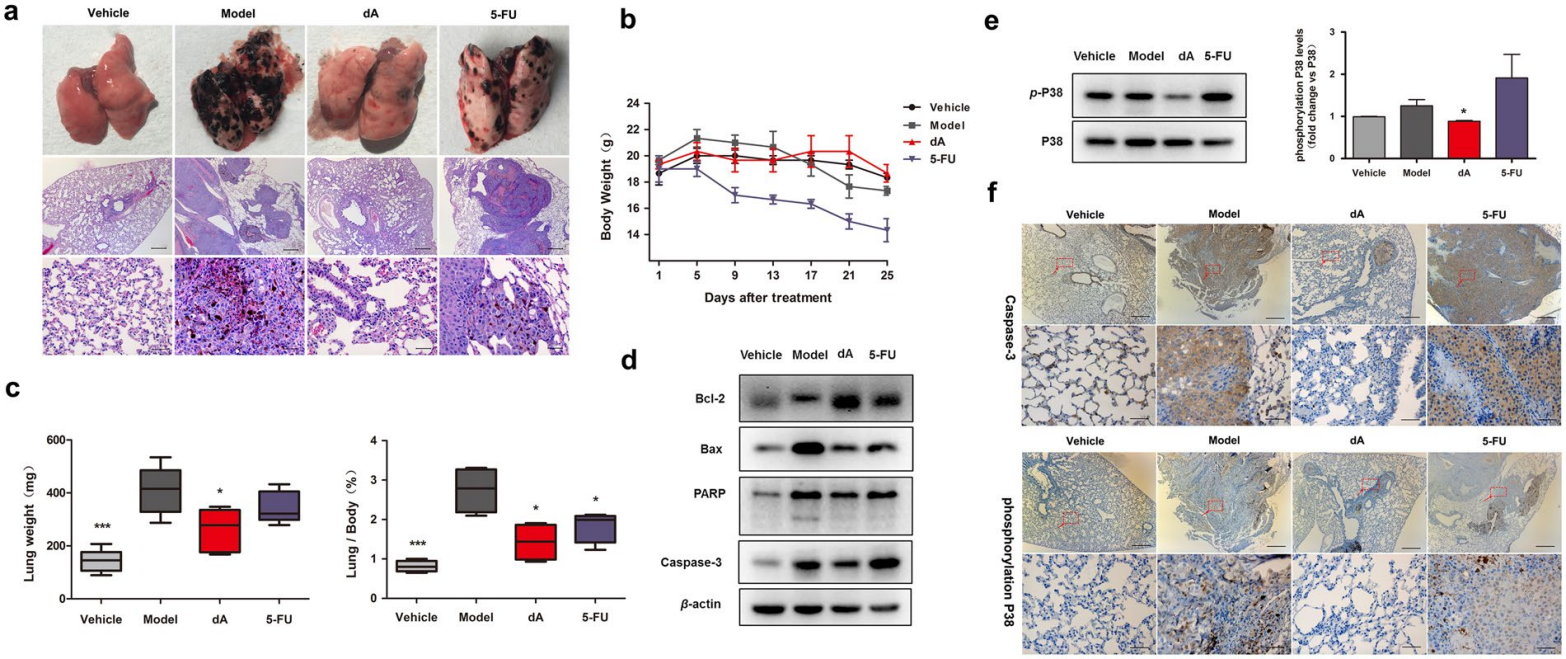
dA inhibited melanoma lung metastasis related to a p38 mediated mitochondria associated apoptosis *in vivo*. The experimental lung metastasis model was producted as follows: 1 × 10^6^ B16F10 cells were injected intravenously via the tail vein and the mice were randomly grouped. Mouse in each experimental group was administrated intraperitoneally injection of dA 50 mg/kg or 5-FU 30 mg/kg, vehicle and model group were subcutaneously injected with saline once a day. (**a**) Lung metastatic nodules were visualized and metastases count at day of sacrifice in lung of each group using H&E staining. Middle panel: scare bar 100 μm; lower panel: scare bar 20 μm. (**b**) The body weight in each group at indicated time point. (**c**) The weight of lungs and the lung/body coefficients in each group. (**d**) Western blot analysis about mitochondria apoptosis proteins as well as the phosphorylated p38 changes (**e**) in lung tissues per experimental group. Full-length blots were presented in Supplementary Fig. 5b. (**f**) Immunohistochemical assay to determine pro-apoptotic proteins: active caspase-3 and p-p38 in lung metastatic models. Scare bar: 100 μm, lower panel represented red box area arrowheads, scale bar: 20 μm. *P* values for comparison of two groups were determined by 2-tailed Student’s t test, *p < 0.05; **p < 0.01; ***p < 0.001 vs Model control.

Metastasis is the central cause lead to mortality in melanoma^42^. We next analyzed the metastatic spread from melanoma *in vivo* by inoculating B16F10 cells intravenously into C57Bl/6J mice. Then these mice were treated by intraperitoneal administration with saline, dA or 5-FU for 24 days. As shown in Fig. 6a,c, the number of lung metastatic nodules as well as the lung weight and lung/body significantly decreased in dA-treated group. Also, the body weights of the mice in dA-treated group were observed to have no significant changes compared with the model group. While, it may be noted that the body weights of mice treated with 5-FU were decreased (Fig. 6b). The data indicated that dA appeared more merit as an anti-metastasis agent with lower side effects compared with 5-FU *in vivo*.

Lung metastasis can be identified as a severe lung injury resulted from apoptotic death of normal lung cells. Caspase family was closely related to the lung injury^43–45^. Our study found that the model group with tumour cells invasion occurred a serious lung injury with high expression of apoptotic proteins, including Bax, PARP and caspase-3 as shown in Fig. 6d and Supplementary Fig. 5b. Treatment with dA significantly protected the normal lung tissue from apoptosis by upregulating Bcl-2 and downregulating Bax, PARP and caspase 3 in lung tissue. Consistent with the *in vitro* results, the anti-metastatic activity of dA was also regulated by p38 activation as shown in Fig. 6e and Supplementary Fig. 5b. Moreover, immunohistochemistry analyses confirmed that dA treatment could decrease the expression of apoptotic proteins in lung tissues, such as active caspase-3 and phospho-p38, compared with the model group (Fig. 6f). Overall, these data suggest that dA inhibited melanoma tumour metastasis by inhibiting tumour cell proliferation in lung tissue, protecting normal lung tissue from apoptosis, which mainly due to the participation of caspase/p38 MAPK.

### dA suppressed tumour growth with a low toxicity *in vitro* and *in vivo*

From the body weights evaluation in per experimental group, we did not observe significant changes in dA group relative to the untreated group (Figs 5c and 6b). Microscopic examination analysis of main organs in mice did not found any pathological changes after treated with dA *in vivo* (Supplementary Fig. 6b), also LO2 and HK-2 cell lines treated with dA found no marked cytotoxicity *in vitro* compared with the vehicle control group (Supplementary Fig. 6a). Therefore, this study indicated that dA with a low toxicity was a potential agent for inhibiting melanoma growth and metastasis.

**Figure 7:**
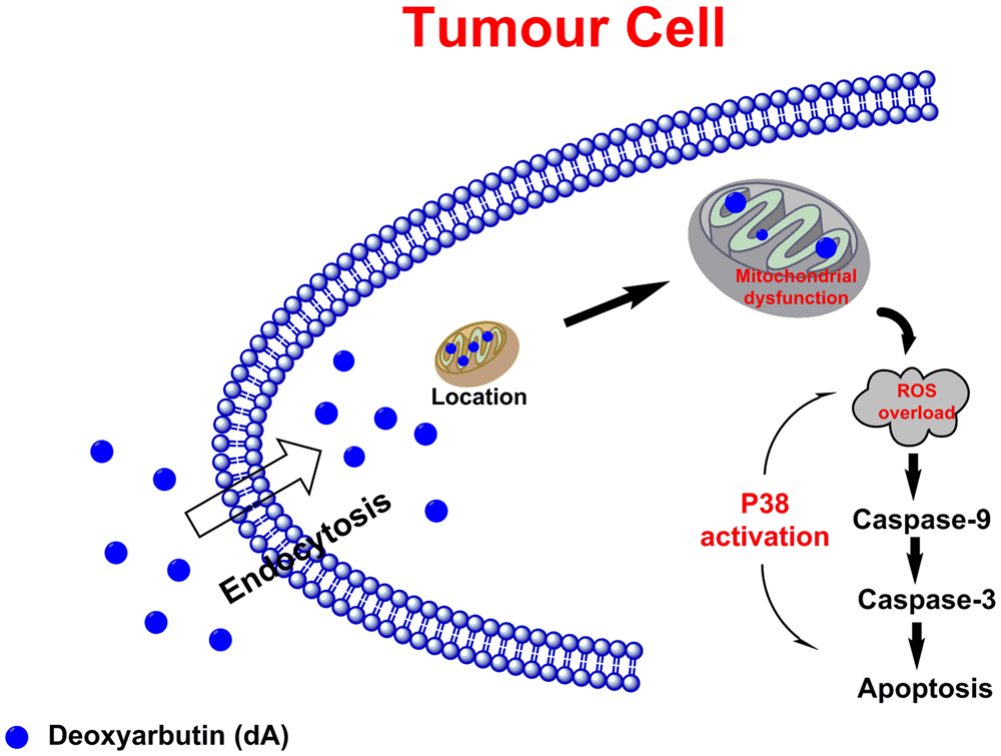
Mechanism schema of dA’antitumour activity. dA could significantly reduce the growth of melanoma *in vitro* and *in vivo* by targeting the mitochondria and initiating the p38 mediated mitochondria associated apoptotic signaling pathway.

Taken together, our *in vitro* and *in vivo* results both indicated that dA played an inhibitory role in B16F10 mouse melanoma progression, and thus increasing animal survival. dA triggered the death process of tumour cells related to mitochondria mediated apoptotic pathway. The signaling events in dA-induced pro-apoptotic effects were vitally interrelated with the up-regulation of caspase/p38 and network that, in turn, activate the intrinsic programed cell death pathway (Fig. 7).

## Discussion

Apoptosis is a major route to deracinate cancer cells and involves cell cycle arrest^45^. Melanoma is a disease characterized by high lung metastases^46^. In this study, we firstly provided that dA could significantly inhibit the growth of melanoma cells *in vitro* and B16F10 xenografts as well as pulmonary metastasis *in vivo*. Our results also elaborated detailed mechanistic of dA’s antitumour action on melanoma cancer (mitochondrial dysfunction, caspases-family activation).

It is well known that mitochondria play an important role in the intrinsic apoptotic pathway. Decrease of mitochondrial membrane potential is one of the earliest events in apoptosis^47^, which causes mitochondrial ATP depletion and ROS generation and then initiates the mitochondria mediated apoptosis signaling. In this study, we observed that dA treatment could lead to mitochondrial swelling in B16F10 cells. We also found dA induced a pronounced increase in ROS from mitochondria, caused a persistent decrease in ATP levels and a significant loss in mitochondria membrane potential. The data suggest that dA’s pro-apoptotic process is closely linked with mitochondrial dysfunction.

The mitochondrial dysfunction triggered the apoptotic signal involving the participation of Bcl and caspase family proteins. Bcl-2 and Bax as well as caspase-9/3were main regulators among the mitochondria related apoptosis^6, 7^. Caspase-9 activation is initiated by mitochondrial dysfunction signals, thus lead to PARP excitation, and finally induce caspase-3 degradation^47^. Our study showed that dA increased the activation of caspase-9/3 and PARP, upregulated the Bax/Bcl-2 ratio, and caused DNA damage, suggesting that the tumour death trigged by dA was related to mitochondria associated apoptotic pathway.

Experimental and clinical evidences have indicated that mitogen activated protein kinase (MAPK) plays a key role in regulating cell apoptosis, and MAPK is activated in 90% of melanomas^48^. It has been reported that p38 MAPK is a pivot in controlling apoptosis, regulating cell cycle progression and cell proliferation^49^. Ji *et al*. found that the expression of Bcl-2 family could be affected by p-p38 levels^50^. Woo *et al*. explained thymoquinone could exert an anti-proliferative and pro-apoptotic effects on mitochondrial signaling by regulating p38 *in vitro* and *in vivo* ^51^. p38 MAPK has been also reported to up-regulate p16 expression, which successively regulate cell cycle genes and ROS levels by inducing mitochondrial dysfunction^49, 51^. Our study confirmed that the activation of p38 was closely associated with the dA-induced apoptosis. Some literatures have reported that dA and its derivatives could regulate ROS levels to light skin or ameliorate hyperpigmented lesions^52^. Our study for the first time proved that dA could target mitochondria, enhance ROS production, induce p38 activation and initiate mitochondria associated apoptotic pathway in melanoma *in vitro* and *in vivo*.

As we known, the worst instincts of melanoma metastasis is one of the greatest challenges in melanoma therapy. Available treatment, such as surgical interventions and radiation, is ineffective in restraining metastasis^53, 54^. In this research, we found that dA dramatically inhibited the metastasis of melanoma cells in mice models as well as antitumour growth via a p38-mediated mitochondria associated apoptotic pathway. Thus, it was clear that dA with satisfying safety could be a novel candidate for metastatic melanoma treatment *in vitro* and *in vivo*.

Our present study declared that mitochondrial dysfunction affects ATP utilization in B16F10 cells. We also found that dA could enrich in mitochondria to induce B16F10 cell apoptosis. Since ATP is one of the main products from the mitochondria during glycolysis^55^, the future research will be required to investigate the relationship between apoptosis and glycolysis induced by dA.

In conclusion, dA suppressed the growth of melanoma *in vitro* and displayed antitumour and anti-metastasis of melanoma *in vivo* with two tumour-bearing mice models. dA could target mitochondria and lead to p38 mediated mitochondria associated apoptotic signaling. Thus, dA has an ideal antitumour property and will be a potential candidate for us to develop a novel antitumour strategy in melanoma treatment.

## Methods

### Reagents

Deoxyarbutin (4-[(tetrahydro-2H-pyran-2-yl) oxy] phenol, purity >99%) was purchased from Sigma (St. Louis, MO, USA). Culture medium (RPIM 1640 medium, DMEM medium), fetal bovine serum (FBS), penicillin (100 U/mL) and streptomycin (100 mg/mL) were purchased from GIBCO (Life Technologies, Carlsbad, CA, USA). Hoechst 33258, Annexin V/PI apoptosis kit, Cell arrest detection kit, ATP detection kit and BCA Protein assay kit were purchased from Beyotime Institute of Biotechnology (Shanghai, China). Mito-sox detection kit, JC-1 detection kit, Mito-tracker and ER-tracker were purchased from Invitrogen Life Technology (Grand Island, NY, USA). 5-FU and molecular probes dansyl chloride (DNS) were purchased from Sinopharm Chemical Reagent Co., Ltd (Shanghai, China). Unless indicated otherwise, the other reagents were purchased from Sigma. dA was dissolved in ethanol and diluted with fresh medium to achieve the desired concentration. The final concentration of ethanol did not exceed 0.1% in the fresh medium.

### Cell culture

Murine melanoma B16F10 cell line and HK-2 human proximal tubule epithelial cell line, LO2 human liver cell line, HS68 and NIH/3T3 fibroblast cell line, Human Umbilical Vein cell line (HUVEC), Human lens epithelium cell line (HLECs), Murine lung carcinoma LL/2 cell line were recipient from Lab of Transplant Engineering and Immunology, Regenerative Medicine Research Center, West China Hospital. The HK-2, HS68, NIH3T3, HUVEC, HLECs and LL/2 cell lines were cultured in DMEM, the B16F10, L02 cell lines were maintained in RPMI-1640 medium supplemented with 10% fetal bovine serum (FBS), penicillin (100 U/mL) and streptomycin (100 mg/mL). Cells were sustained at 37 °C in a humidified atmosphere with 5% CO_2_.

### Cell viability assay

Cell viability was measured by using a CCK-8 kit (Dojindo, Tokyo, Japan) or MTT assay (Sigma, USA), according to the manufacturer’s instructions. Briefly, cells were plated at a density of 5 × 10^3^ per-well into 96-well plates. Once the confluence reached to 80%, cells were incubated with different concentrations of dA (5, 10, 50, 100 and 200 μM) for 24 h. At the endpoint, with 10 μL CCK-8 solution or MTT 0.5 mg/mL for further 3 h, then the absorbance at 490 nm was measured by using a SpectraMax M5 microplate reader (Molecular Devices, LLC, Sunnyvale, CA, USA). The percentage cell viability was presented as absorbance of the experiment samples/absorbance of the control without treating dA × 100%.

### Colony formation assay

Colony formation ability assay was performed as previously described^56^. B16F10 cells were re-plated in specified numbers (100 cells/well) in 6-well plate, and treated with various concentrations of dA (0–200 μM). Then, the cells were incubated with fresh medium of dA once every 3 days for additional 13 days and stained with 0.5% crystal violet after 4% paraformaldehyde fixing. Then, the morphology of cells was observed by a microscope (uX71; Olympus Corp., Tokyo, Japan).

### Cell cycle and apoptotic assays

Cell cycle distribution was analyzed following dA treatment by using flow cytometry. In brief, B16F10, NIH/3T3 or HS68 cells were treated with 50 μM dA for 24 h, harvested, washed once with phosphate buffered saline (PBS, Solarbio Science & Technology Co., Ltd, Beijing, China) and fixed using 75% ethanol at 4 °C overnight. Following centrifugation at 800 g for 5 min, the cells were then resuspended and stained by a cell cycle analysis kit, according to the manufacturer’s instructions. The cells were analyzed by using a flow cytometer (Becton Dickinson, USA). Finally, all the data was analyzed by Flow Jo software. Cell apoptosis was analyzed by using Annexin V-FITC staining. Briefly, cells were harvested after treated with 50 μM dA for 24 h, and washed once with PBS followed by stainning with Annexin V-FITC at 37 °C for 15 min. The data were obtained from flow cytometer detection. Viable cells were negative for both PI and annexin V-FITC; nonviable cells underwent necrosis, were positive for PI, but negative for Annexin V-FITC; early apoptotic cells were positive for Annexin V-FITC but negative for PI, while late apoptotic cells both labeled strong Annexin V-FITC and PI.

### Morphological analysis

After incubating with 50 μM dA for 24 h, B16F10 cells were washed with PBS and fixed with 4% paraformaldehyde for 20 min at room temperature. Then the fixed cells were subsequently stained with DAPI (5 μg/mL) at 37 °C for 15 min, washed with PBS twice and the changes in the nuclei were examined using fluorescence microscope (Zeiss, Axiovert 200, Germany).

### Transmission electron microscopy

B16F10 cells or NIH/3T3 cells treated with dA (50 μM) for 24 h were fixed with 75% ethyl alcohol in 4 °C overnight. Then the morphological changes in the ultrastructure were performed by TEM as previously described^57^. The fixed-cells were post-fixed with 1% osmium tetroxide, and dehydrated in acetone solutions, followed by infiltrating in Epox 812 (Zhongjingkeyi Technology Co., Ltd., Beijing, China) for 3 h and then embedded. Semi-thin sections (0.6~0.8 μm) were cut and ained with methylene blue (Sigma-Aldrich). The ultra-thin sections (<0.1 μm) used for observation) were cut using a diamond knife (Zhongjingkeyi Technology Co., Ltd., Beijing, China), and stained with lead citrate and uranyl acetate. Sections were then examined by using a transmission electron microscope (H-600IV, Hitachi, Tokyo, Japan).

### Intracellular location assay

To assess the localization of dA in cells, we applied DNS to label dA and incubated with B16F10 cells and NIH/3T3 cells. Briefly, cells were seed in 24 well plates, followed by 2 h or 24 h treatment with DNS-labeled dA. Then the cells were incubated with Mito-tracker or ER-tracker for 30 min. DAPI were used to stain the nuclei. Followed by PBS washing twice and the fluorescence image were obtained by using confocal microscope (Zeiss, Axiovert 200, Germany).

### Measurement of intracellular mitochondrial function in B16F10 cells

To assess the intracellular mitochondrial function in cells, ROS, ATP levels and mitochondrial membrane potential were detected as previously described^58^. Briefly, B16F10 cells were treated with 50 μM dA for 24 h and washed with RPMI-1640 medium twice and then incubated with 5 μM Mito-sox or ATP detection reagent at 37 °C for 30 min for ROS and ATP detection. Then, the fluorescence intensity was measured by a multi-mode microplate reader (BioTek, SYNERGY IMX, USA) according to the manufacturer’s instructions. The ratios of fluorescence intensity to protein concentration were calculated to normalize the results. Each group was acquired more than 20000 individual cells. The JC-1 probe was used to measure the mitochondrial membrane potential. B16F10 cells treated with dA of 50 μM for 24 h, then, the cells were loaded with 2 mg/L of JC-1 at 37 °C for 15 min. Then the fluorescence intensity was analyzed by a confocal microscope (Zeiss, Axiovert 200, Germany).

### Western blotting

B16F10 cells in 6-well plate were collected and washed with PBS and then lysed with 60 μL lysis buffer (Beyotime) for 15 min at 4 °C. The cell supernatant was collected by centrifugation at 12000 g centrifuged for 10 min at 4 °C. Protein concentration was determined using the BCA protein assay kit. The equal amount (30 μg) of extract protein was loaded, separated by 15% SDS-PAGE, and transferred to a polyvinylidene difluoride membrane (PVDF, Carlo Erba reagents, Milan, Italy). After blocked with TBST containing 5% skimmed milk for 1 h at 37 °C, the membrane was incubated with the following primary antibodies at 4 °C overnight: anti-caspase3/active-caspase 3 (1:1000), from Cell Signaling Technology (CST, Danvers, MA, USA); anti-caspase 9 (1:1000), anti-Bcl-2/Bax (1:1000), anti-PARP (1:1000), anti-P38 MAPK (1:1000), anti-phospho-P38 MAPK (1:1000), anti-GAPDH (1:1000), from CST; anti-VDAC (1:500) from Abcam (Cambridge, MA, USA). After incubation, the membrane was washed with TBST for 3 times and incubated with secondary rabbit or mouse antibodies (Dako, Danish) for 1 h at room temperature. Following a second wash, the separated protein bands were visualized using Immobilon-Western Chemiluminescence HRP substrate (Millipore, USA) and analyzed by using ChemiDoc™- XRS imaging system (Bio-Rad). Quantifications of relative protein expressions were carried out by using the Image Lab 3.0 software (Bio-Rad).

As for proteins obtained from animal tissue, the tumour sections were washed with PBS and then lysed with lysis buffer (Beyotime, 1000 uL/100 mg) for 30 min at 4 °C. Then the lysed supernatant was collected according to the above methods.

### Experimental melanoma tumour model

Male C57Bl/6 J mice (16 weeks old) with body weights ranging from 20–25 g were purchased from Chengdu Dossy experimental animal Co. Ltd. (Chengdu, China) and the experimental melanoma tumour model was conducted as described previously^59^. The animal room was controlled to maintain temperature (22 ± 2 °C), light (12 h light/dark cycles) and humidity (50 ± 10%). After 1 week of acclimatization, the mice were depilated and implanted in the right flank region with the B16F10 cells (1.0 × 10^6^). Seven days following implantation, the mice with tumour sizes >50 mm^3^ were selected and randomly divided into three groups (n = 10 per group), termed the model, dA and 5-FU groups. These groups were treated with saline, 50 mg/kg dA and 30 mg/kg 5-FU respectively, the concentrations of which were determined in a preliminary study. Equal volumes of the drugs and vehicle were administered orally to the mice every day for 3 weeks. The body weight and tumour size were measured three times weekly and the tumour volumes were calculated according to the following formula: width^2^ × length × 0.5. Ethical approvals for all experiments and methods had been obtained from the Ethics Committee of West China Center of Medical Sciences, Sichuan University (Approval 2014003B), and were performed in accordance with ARRIVE guideline^60^.

### Experimental melanoma metastasis model

40 male C57Bl/6J mice were purchased from Chengdu Dossy experimental animal Co. Ltd. (Chengdu, China). After 1 week of acclimatization, 30 male C57Bl/6J mice were injected intravenously via the tail vein with 1 × 10^6^ B16F10 cells to produce experimental lung metastasis according to the previous report^56^, and then randomly divided into three groups (n = 10): model group, dA group and 5-FU (positive control) group. 10 male C57Bl/6J mice without B16F10 cells injection were used as vehicle control. Animals in each group were intraperitoneally injected with dA 50 mg/kg, 5-FU 30 mg/kg or saline as vehicle in normal and model group once daily. All animals were killed on the 24th day, followed by a comprehensive visual examination of all organs. Black dots on lung surface were counted and confirmed as melanoma metastases. Mouse tissue samples (heart, liver, spleen and kidney) were fixed in 4% paraformaldehyde and embedded in paraffin. The tissue samples were processed into sections of 4 μm thick sections and the slides were stained with hematoxylin and eosin (H&E) according to standard protocols. Ethical approvals for all experiments and methods had been obtained from the Ethics Committee of West China Center of Medical Sciences, Sichuan University (Approval 2014003B), and were performed in accordance with ARRIVE guideline^60^.

### Immunohistochemical staining

Immunohistochemical staining for activated caspase-3 and phosphorylated p38 were carried out as follows as described previously^56^. Tissue sections removed from the mice in per experimental group were washed in dH_2_O, and after antigen retrieval by using 10 mM citrate buffer (pH 6.0) in EZ antigen retriever for 10 min of 2 times, endogenous peroxidase activity was then blocked by 3% H_2_O_2_ for 20 min. Each tissue section was blocked with incubating in 10% normal goat serum for another 30 min. Then the primary antibody (active caspase-3 and p-p38) was added onto the slices at 4 °C overnight. Subsequently, the above slices were incubated with peroxidase conjugated second antibody (HRP-labelled goat anti-rabbit IgG antibody, ZSGB-Bio Origene, Beijing, China) for 30 min. After washing with PBS, coloring reaction was executed. Sections were then examined by using a microscope (uX71; Olympus Corp., Tokyo, Japan).

### Data and statistical analyses

Data are presented as means ± S.D. Student’s paired t-tests and ANOVA were used to analyze differences in cytotoxicity, ROS, ATP levels, tumour weight, body weight, lung tumour metastases, and so on. *P* values < 0.05 were considered statistically significant.

## Electronic supplementary material

Supplementary Information
