# Supplementary material for: Deoxyarbutin displays antitumour activity against melanoma in vitro and in vivo through a p38-mediated mitochondria associated apoptotic pathway
Source: Sci Rep. 2017 Aug 3;7:7197. doi: 10.1038/s41598-017-05416-8 (PMC5543205; doi:10.1038/s41598-017-05416-8)
Supplement: Supplementary file 1 — Supplementary Information [file 41598_2017_5416_MOESM1_ESM.pdf]

Deoxyarbutin displays antitumour activity against melanoma *in vitro* and *in vivo* through a p38-mediated mitochondria associated apoptotic pathway

Limei Ma[1+], Yao Xu[1+], Zeliang Wei[1], Guang Xin[1], Zhihua Xing[1], Hai Niu[2]* and Wen Huang[1]*

1 Laboratory of Ethnopharmacology/Regenerative Medicine Research Center, West China Hospital, Sichuan University, Chengdu, Sichuan 610041, China
2 College of Mathematics, Sichuan University, Chengdu, Sichuan 610041, China
* First corresponding author: Wen Huang, huangwen@scu.edu.cn
* Second corresponding author: Hai Niu, niuhai@scu.edu.cn
+ These authors contributed equally to this work

Supplementary Information

Cell line	Origin	Species	EC50（μM）	Assay
NIH/3T3	fibroblasts	mouse	122.80	MTT/CCK-8
HS68	fibroblast	human	108.34	MTT/CCK-8
B16F10	melanoma	mouse	39.56	MTT/CCK-8
LL/2	lung carcinoma	mouse	44.69	MTT
LO2	liver	human	1.43×10^{3}	MTT
HK-2	proximal tubule	human	2.56×10^{3}	MTT
HUVECs	umbilical cord	human	6.20×10^{3}	MTT
HLECs	lens epithelium	human	5.56×10^{3}	MTT

Supplementary Figure 1. Parameters of dA-induced inhibition of cell viability on various cell lines. EC50 (μM) = concentration required to produce 50% of the effects. MTT or CCK-8 assays were performed treating cells for 24 h in the absence (Vehicle) or in the presence of increasing concentrations of dA , n = 3.

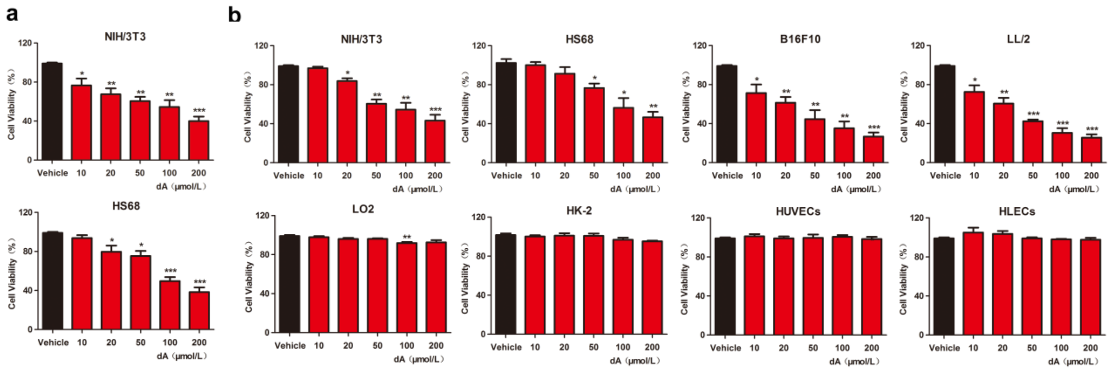

Supplementary Figure 2. Inhibition of cell viability induced by dA on various cell lines by CCK-8 and MTT assays. Cell viability was determined by CCK-8 assay (**a**) and MTT assay (**b**) after 24 h treatment in the absence (Vehicle) or in the presence of increasing concentrations of dA (10, 20, 50, 100 and 200 μM) on NIH/3T3, HS68, B16F10, LL/2, LO2,HK-2, HUVECs and HLECs cell lines, n= 3.

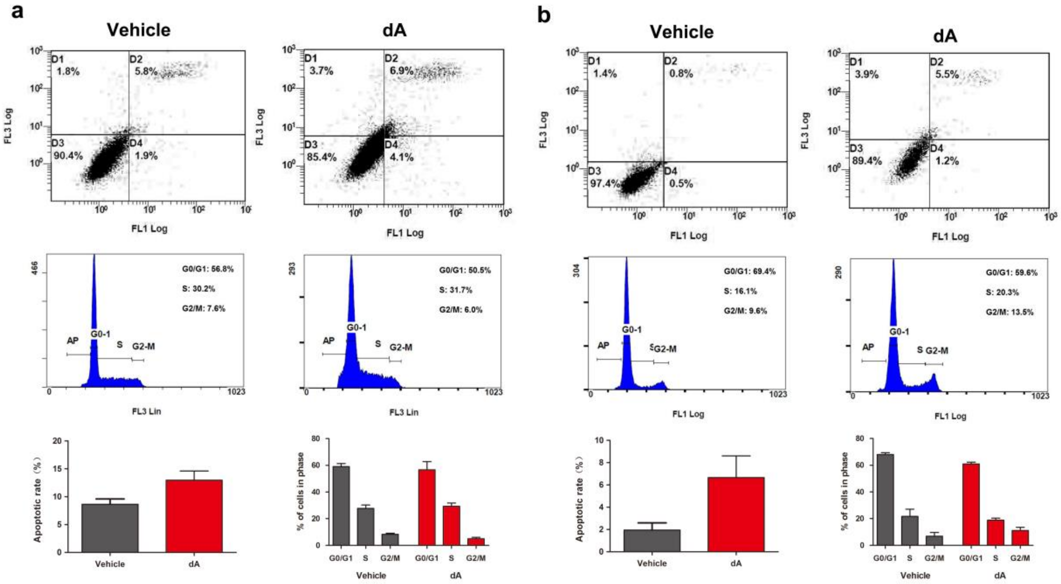

Supplementary Figure 3. Apoptosis and cell arrest in NIH3T3 (a) and HS68 (b) cells after treating with dA. Cells were cultured in the presence of 50 μM dA or Vehicle (control) for 24 h. Flow cytometry evaluation was used to make quantitative analysis.Cells were stained by Annexin V/PI for apoptotic detection. DNA cycle arrest was detected by PI staining. The lower panel shown represents the apoptotic rate and cell numbers in indicated period of cell cycle. Images and data are representative of 3-5 independent experiments.

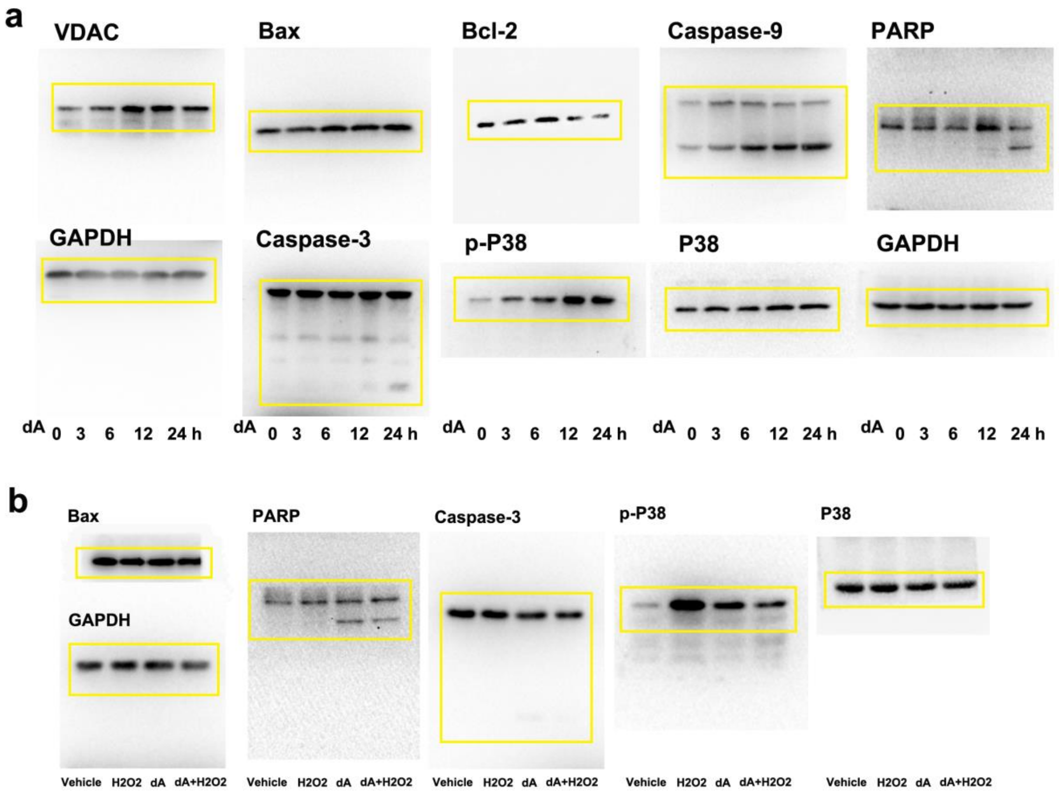

Supplementary Figure 4. Illustrated the full length western blots of the cropped blots

shown in Fig. 4.** (**a**) Represented the full length of indicated proteins in Fig. 4a,b,c. (**b**) Indicated the full length of proteins shown in Fig. 4d.e,f. The hatched yellow lines delineate the correct sized bands for each protein.

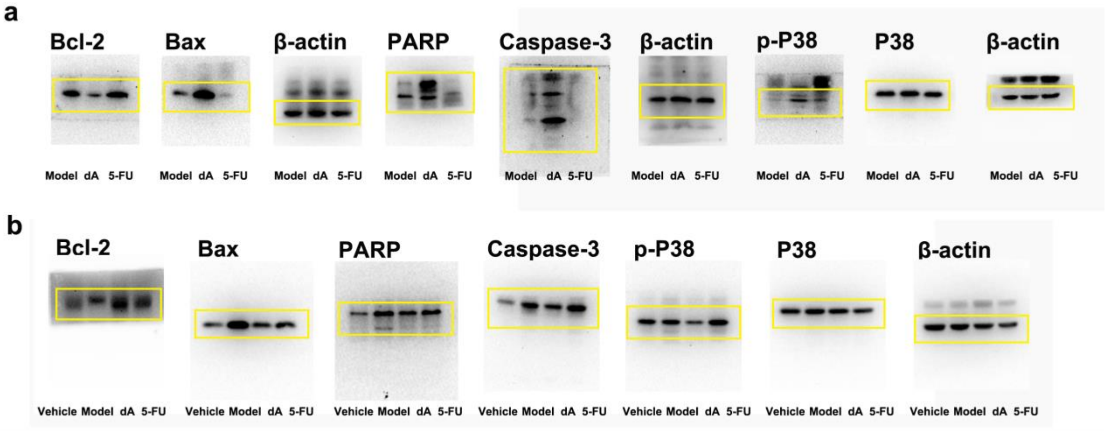

Supplementary Figure 5. Declared the full length of the cropped blots shown in Fig. 5 and Fig. 6. (**a**) Represented the full-length blots of the proteins obtained from the mice in experimental melanoma tumour model shown in Fig. 5. (**b**) Indicated the full-length blots of the proteins in experimental melanoma metastasis model mice shown in Fig. 6. The hatched yellow lines delineate the correct sized bands for each protein.

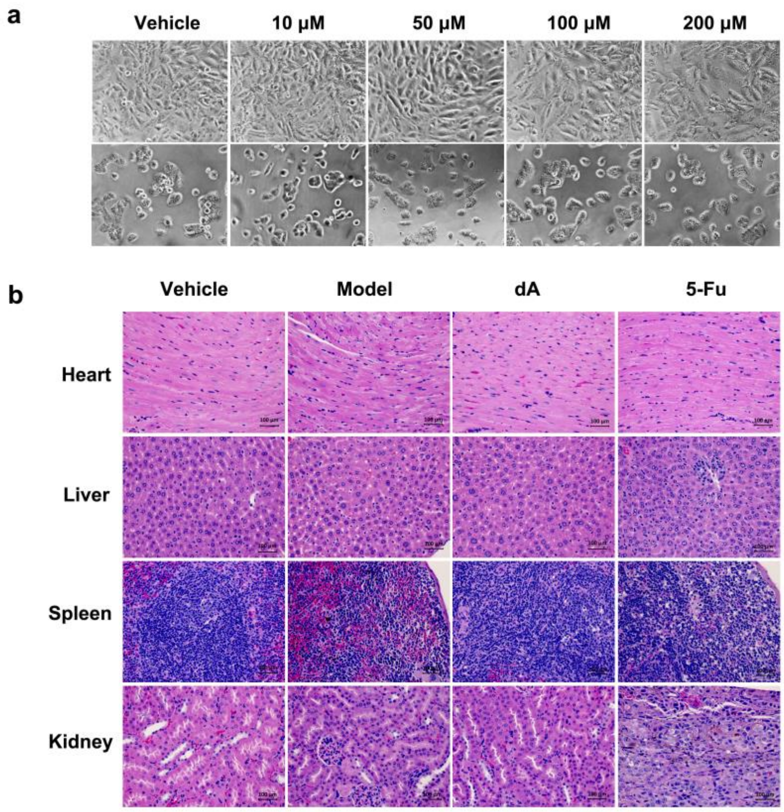

Supplementary Figure 6. dA suppressed tumor growth with a low toxicity *in vitro* and *in vivo*. (**a**) HK-2 cells (above line) and LO2 cells (below line) were incubated in dA for 24 h,

concentration varies from 10 μM to 200 μM. Vehicle indicated cells without treating dA. (**b**) H&E stained main organs of mice from per experimental group in lung metastatic model sets to evaluate the toxicity of dA and 5-FU used in our experiment. Vehicle indicated the mice given saline, Model group indicated the mice with lung metastasis given saline. Images are representative of 3 independent experiments.
